# Ecdysteroids: production in plant in vitro cultures

**DOI:** 10.1007/s11101-016-9483-z

**Published:** 2016-11-24

**Authors:** Barbara Thiem, Małgorzata Kikowska, Michał P. Maliński, Dariusz Kruszka, Marta Napierała, Ewa Florek

**Affiliations:** 1 Department of Pharmaceutical Botany and Plant Biotechnology, Poznan University of Medical Sciences, 14 Św. Marii Magdaleny Str., 61-861 Poznan, Poland; 2 Department of Toxicology, Poznan University of Medical Sciences, 30 Dojazd Str., 60-631 Poznan, Poland

**Keywords:** Ecdysteroids, Elicitation, In vitro cultures, Plant biotechnology, Precursor feeding

## Abstract

Ecdysteroids are secondary metabolites, widely distributed in the animal and plant kingdoms. They have a wide range of pharmacological effects in vertebrates, including mammals, most of which are beneficial for humans. Therefore, they have become compounds of interest for the pharmaceutical industry due to their adaptogenic, anabolic, hypoglycaemic, hypocholesterolaemic and antimicrobial activities, which are still being researched. Nowadays, ecdysteroids are present as active ingredients in bodybuilding supplements. Because of their complex structures, their chemical synthesis seems unprofitable and impractical. Due to high content of ecdysteroids in many plants, they are primarily obtained by extraction of the plant material. Plant in vitro cultures provide an alternative source of these compounds, helping to avoid problems associated with field production—such as variable yield or dependence on environmental factors, as well as limited availability of natural resources. Plant cell and tissue cultures may be suggested as alternatives for the production of plant biomass rich in pharmaceutically active ecdysteroids. Moreover, the use of common biotechnological strategies, such as elicitation or precursor feeding, may further increase the yield and improve production of these compounds. In this paper, we describe general information about ecdysteroids: their structure, biosynthesis, distribution, role in plants, and we review recent studies on micropropagation of ecdysteroid-producing plants and cell cultures, and potential ability of ecdysteroids enhancement in in vitro cultures.

## Introduction

Ecdysteroids, triterpenoid compounds playing vital roles in arthropods and plants, are a group of chemicals with interesting biological activities in mammals that should be furtherly elucidated (Dinan 2001; Lafont and Dinan 2003; Dinan and Lafont 2006; Speranza 2010). Due to limited availability of these compounds from natural sources and unprofitable chemical synthesis, plant in vitro cultures offer biomass production rich in ecdysteroids. Generally, plant tissue cultures are a biotechnological technique highly regarded for its usefulness as a sustainable source of genetically uniform plant material and the possibility of rapid, clonal propagation of plants, regardless of environmental factors. Uniformity of the plant material also ensures the uniformity of its chemical composition. In case of medicinal plants, it is particularly important to provide a homogenous source of chemicals with biological activity. Less known chemicals produced by rare or endangered plants can be therefore obtained from plant tissue cultures and their activity can be studied (Karuppusamy 2009). This review summarizes examples of in vitro ecdysteroid production and presents diverse techniques that can significantly increase their yield.

## The structure and biosynthesis of ecdysteroids

**Fig. 1:**
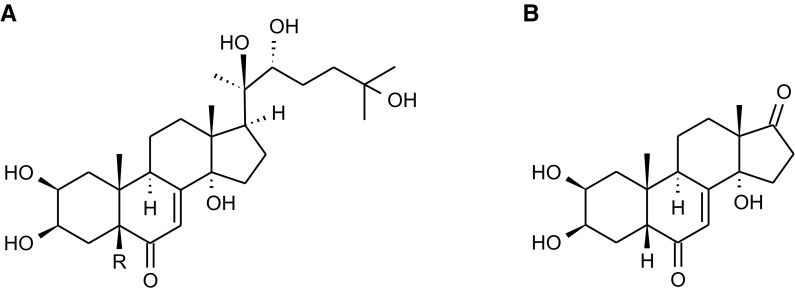
Structures of the most common ecdysteroids A 20-hydroxyecdysone (R=H) and polypodine B (R=OH) B rubrosterone, an ecdysteroid without aliphatic side chain

**Fig. 2:**
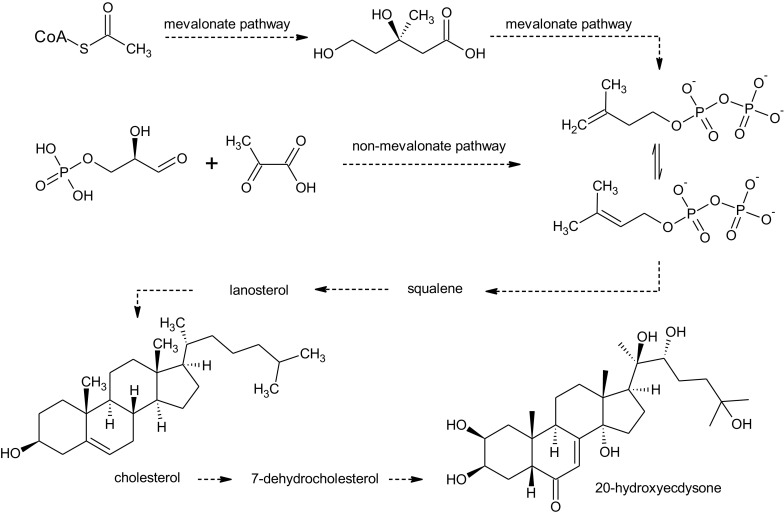
The synthesis of 20-hydroxyecdysone

Ecdysteroids are sterols chemically related to triterpenoids and therefore share most steps of the biosynthetic pathway with cholesterol, which is also considered their direct precursor. The important intermediates are isoprenoid units IPP (isopentenyl pyrophosphate) and DMAPP (dimethylallyl pyrophosphate), which typically are the products of mevalonate pathway, starting from acetyl-CoA as the basic building block. Higher plants are also able to synthesize isoprenoids from pyruvate and glycerylaldehyde-3-phosphate (via G3P-Pyr or non-mevalonate pathway). Thereafter, isoprene units condensed to squalene undergo epoxidation and cyclization to lanosterol. As the majority of ecdysteroids is based on C27 cholest-7-en-6-one backbone, subsequent steps of their biosynthesis include conversion to cholesterol, dehydrogenation to 7-dehydrocholesterol and further molecular modifications, and, most importantly, hydroxylation at various carbon atoms (Dinan 2001; Baltaev 2000; Festucci-Buselli et al. 2008; Ikekawa et al. 2013). Typical ecdysteroids are 20-hydroxyecdysone (20E) and polypodine B (polB) (Fig. 1). In ecdysteroids such as rubrosterone, the aliphatic side chain attached to D ring is cleaved, leaving a carbonyl group (Fig. 1). The synthesis of 20-hydroxyecdysone is shown in Fig. 2.

Relating to the scope of this article, it has to be noted that a significant portion of knowledge on the biosynthesis of ecdysteroids has been acquired thanks to research conducted on plant in vitro cultures (Fujimoto et al. 2000; Ohyama et al. 1999; Okuzumi et al. 2003).

## The diversity and distribution of ecdysteroids

In both plants and animals, the most predominant ecdysteroids are 20-hydroxyecdysone (20E) and polypodine B (polB) (Dinan and Lafont 2006). Screening research on numerous species of plants revealed a considerably greater diversity of ecdysteroid derivatives in plants than in arthropods. While insects produce mostly 20-hydroxyecdysone and alpha-ecdysone as physiological hormones, a single species of a plant typically contains 20-hydroxyecdysone as the major component and a complex mixture of structurally-related ecdysteroids in small quantities (Báthori et al. 1999; Festucci-Buselli et al. 2008).

The most common derivatives contain additional hydroxyl groups. Deoxy-derivatives and compounds containing additional methyl groups are also found. The presence of numerous hydroxyl groups enables the occurrence of diverse ecdysteroid conjugates, such as acetate and benzoate esters, glycosides and other various derivatives, such as acetonides. Derivatives in which the side chain is cyclized into a lactone or acetal are also reported. Another class of ecdysteroids is represented by rubrosterone, containing a carbonyl group instead of aliphatic side chain (Baltaev 2000; Festucci-Buselli et al. 2008; Mamadalieva 2012; Mamadalieva et al. 2014).

## The effects of ecdysteroids in mammals

Because of their disruptive effects on insect physiology, ecdysteroids were considered to be used as insecticides, but any harmful effects on mammals had to be excluded. Research has shown that the toxicity of ecdysteroids in mammals is very low, with LD50 for 20-hydroxyecdysone estimated above 6 g kg^−1^ of body mass. One of the first pharmacological effects attributed to ecdysteroids was significant increase of protein synthesis in rat liver polysomes by direct stimulation of translation (Lafont and Dinan 2003; Báthori and Pongracz 2005; Dinan and Lafont 2006; Festucci-Buselli et al. 2008).

Ecdysteroids were reported to affect metabolism of three main groups of nutrients—proteins, carbohydrates and lipids. Ecdysteroids also reduce hyperglycaemia induced by glucagon. Several other effects that have been reported include nephroprotective and hepatoprotective activities, increase in bile secretion and restoration of normal glomerular filtration rate (Lafont and Dinan 2003; Dinan and Lafont 2006; Báthori and Pongracz 2005; Festucci-Buselli et al. 2008; Graf et al. 2014).

Ecdysteroids seem to have beneficial effect on human skin. Apart from improving its condition, ecdysteroids act as inhibitors of skin collagenase and accelerate the healing of small wounds and burns (Detmar et al. 1994; Nsimba et al. 2008).

Ecdysteroids can also influence the activity of the central nervous system. The effects can be partially connected to neurotransmitter metabolism (increased synthesis of GABA, decreased breakdown of acetylcholine), as well as neuromodulatory effects on the GABA_A_ receptor (Dinan and Lafont 2006). Recent reports also suggest that ecdysteroids can protect neurons against harmful effects of various drugs, such as alcohol and benzodiazepines (Xun et al. 1999). Ecdysteroids isolated from *Vitex doniana* Sweet are reported to interfere with monoaminergic neurotransmission, exerting an antidepressant effect in mice (Báthori and Pongracz 2005; Festucci-Buselli et al. 2008; Ishola et al. 2014).

There have been also reported other effects of ecdysteroids, such as improved activity of immunological system in immunodeficient laboratory animals (Shakhmurova et al. 2012), as well as antioxidant, antimicrobial and antiproliferative properties (Báthori and Pongracz 2005; Festucci-Buselli et al. 2008; Mamadalieva et al. 2013).

The multidirectional activity of ecdysteroids has raised the question of how these compounds are able to affect that many processes. A plausible hypothesis is that ecdysteroids affect Akt/PKB (protein kinase B) pathway, an intracellular signaling cascade that plays a central role in mammalian cell metabolism and therefore regulates many phenomena (Lafont and Dinan 2003).

## The role of ecdysteroids in plants

Plants are able to synthesize and accumulate ecdysteroids in incomparably higher quantities than insects. As a result, the possibility that these compounds acted as hormones in plants was rather excluded. It is now known that the most probable role of ecdysteroids is acting as phytoalexins and protecting plants from phytophagous insects and soil-dwelling nematodes (Dinan 2001; Festucci-Buselli et al. 2008). Moreover, physical damage to *Spinacia oleracea* L. roots, as well as treatment with plant stress-signaling molecules (such as jasmonates), induces production of ecdysteroids (Schmelz et al. 1998).

Overall, these data suggest that ecdysteroids are produced by plants in response to general environmental stress factors.

## Present sources of ecdysteroids

The first ecdysteroids of plant origin were discovered in a conifer *Podocarpus nakaii* Hayata in 1966 and named ponasterones A, B and C. Not much later, other ecdysteroids, especially 20-hydroxyecdysone, were found in *Podocarpus elatus* R.Br., a fern *Polypodium vulgare* L. and *Achyranthes faurieri* H. Lev. and Vaniot.—an angiosperm belonging to Amaranthaceae family (Baltaev 2000). These early findings discovered in taxonomically different species sparked an interest among researchers, and during a screening research it soon became evident that occurrence of ecdysteroids was widespread in plant kingdom. Several taxons are particularly rich in these secondary metabolites. As far as ferns are concerned, ecdysteroids were found in more than 50% of the screened species belonging to families Polypodiaceae, Pteridaceae and Blechneaceae (Wu et al. 2010). Ecdysteroids are also present in several conifers and a vast number of angiosperm species belonging to families Caryophyllaceae, Amaranthaceae, Chenopodiaceae, Asteraceae and Lamiaceae. Especially worth mentioning is the genus *Silene* in which diverse ecdysteroids are present in large quantities (Meng et al. 2001). In these plants, ecdysteroids can reach concentrations of ca. 1–2% of the plant’s dry weight. However, there are exceptions within the aforementioned families, even within genera, specified as ecdysteroid-negative species. It is speculated that a majority of plants are theoretically able to synthesize ecdysteroids, but the responsible genes have been silenced, perhaps in favor of the interaction between plants and harmless or beneficial insects (such as pollinators) (Saatov et al. 1994, Lafont and Dinan 2003). Very few of the crop species are ecdysteroid-positive, which limits the possible dietary sources of ecdysteroids. Among the most rich edible species are spinach (*Spinacia oleracea* L.) and quinoa (*Chenopodium quinoa* Willd.) (Dinan and Lafont 2006).

In recent years, many more plants species were found to synthesize ecdysteroids and were thoroughly investigated as to their exact qualitative and quantitative composition. Several plants can be listed here as species of particular interest and the subject of numerous studies, e.g. *Serratula wolffi* Andrae and *S. coronata* L. (Vanyolos et al. 2012; Odinokov et al. 2002). Many *Silene* species, including *S. viridiflora* L. (Simon et al. 2009) and *S. otites* (L.) Wibel (Báthori et al. 1999), were thoroughly investigated due to the significant abundance of ecdysteroids in this taxon (Zibareva et al. 2003; Nowak et al. 2012). A few *Ajuga* species—*Ajuga reptans* L.*, A. remota* Benth. *A. nipponensis* Makino and *A. turkestanica* (Regel) Briq. are also being currently studied as a source of ecdysteroids, revealing characteristic derivatives such as ajugalactone, with a broad spectrum of pharmacological activities (Coll et al. 2007; Calcagno et al. 1995; Cocquyt et al. 2011).

Currently, ecdysteroids for commercial preparations are obtained from ecdysteroid-rich plants that contain them in considerable quantities. The taxons which are especially worth mentioning are those used in traditional medicine: *Ajuga turkestanica*, *Leuzea carthamoides* (Willd.) Iljin., and several *Pfaffia* species in which ecdysteroids are particularly abundant in the roots. Two monocotyledonous species*: Cyanotis vaga* (Lour.) Schult. and Schult.f. and *C. arachnoidea* C.B.Clarke, and several species from ferns belonging to *Polypodium* are used as a source of an ecdysteroid-rich extract (Saatov et al. 1994; Messeguer et al. 1998; Borovikova and Baltaev 1999; Lafont and Dinan 2003).

Many of these plants were also introduced to in vitro cultures, providing both a sustainable source of ecdysteroids, as well as a model for a better understanding of the ecdysteroid biosynthesis and diversity.

## Ecdysteroid production in plant in vitro cultures

Plant tissue culture offers not only a viable alternative method for plant propagation, but has also become an attractive technique in the production of secondary metabolites of pharmaceutical importance. Plant tissue culture is a potential novel approach to obtaining various substances, especially those with complicated structure, relatively high efficiency and low cost (Rout et al. 2000; Verpoorte et al. 2002; Karuppusamy 2009; Filova 2014).

In vitro plant cultures are a widely used technique in the production of bioactive secondary metabolites. Different approaches can be used to increase secondary metabolites production in plant in vitro cultures. Culture media composition, such as nitrogen, phosphate or sucrose concentration, can be changed, just as the addition of plant growth regulators in various doses and ratios. Adjusting temperature, changing illumination and photoperiod, use of various biotic and abiotic elicitors, precursor feeding, etc.—all of the above biotechnological strategies can have a significant effect on secondary metabolite yield. Enhancement of desired compound production by elicitation is one of the few approaches that have recently found commercial application (Smetanska 2008).

Various strategies may be used to induce ecdysteroid overproduction in plant tissue cultures. Some of them are presented in Table 1. Production and accumulation of ecdysteroids by in vitro culture has become of increased interest, as it provides a stable source of these compounds. Levels of ecdysteroids in wild plants may be highly variable, depending on the site of vegetation, harvesting period and many environmental factors that cannot be controlled. In vitro methods allow plant cultures to be grown in controlled laboratory conditions and to produce biosafe metabolites according to good manufacturing practices (GMP) monitored on daily basis. In addition, clonal propagation method can be utilized to overcome natural plant heterogeneity. Types of cultures such as cell suspension culture or root cultures in liquid medium are particularly suitable for manipulation by the biotechnological methods mentioned above, in order to improve and optimize the yield of secondary metabolites (Collin 2001; Smetanska 2008; Karuppusamy 2009).

**Table 1:** Examples of ecdysteroid-producing in vitro systems

Plant species/family	Type of in vitro culture	Best medium and/or treatment for highest metabolites production	Secondary metabolites	Authors
*Achyranthes bidentata* Blume Amaranthaceae	Cell suspension	MS with NAA (1.5 μM) + BAP (1.5 μM) Elicitor: MeJA	20E	Wang et al. (2013)
*Ajuga multiflora* Bunge. *Lamiaceae*	Hairy roots	Hormone free MS	20E	Kim et al. (2005)
*Ajuga reptans* L. Lamiaceae	Shoot and root cultures	MS with NAA (0.5 μM) or IAA (3.6 μM) + BAP (1.6 μM)	Ajugalactone, cyasterone, 29-norcyasterone, sengosterone, 29-norsengosterone, 20E	Tomas et al. (1993)
	Cell suspension	MS with NAA (1.0 mg l^−1^)		Filippova et al. (2003)
	Hairy roots	Hormone free MS		Matsumoto and Tanaka (1991)
*Ajuga turkestanica* (Regel) Briq. Lamiaceae	Cell suspension	B_5_ with 2,4-D (2.3 μM) Precursors: Na(CH_3_COO), MVA, MVL, cholesterol Elicitor: MeJA	Cyasterone, 20E, turkesteron	Cheng et al. (2008)
	Hairy roots	MS with BAP (2.2 μM) + IBA (2.5 μM) Precursors: Na(CH_3_COO), MVA, MVL, cholesterol Elicitor: MeJA		
*Chenopodium album* L. Chenopodiaceae	Cell suspension	MS with 2,4-D (0.165 mg l^−1^) + Kin (0.107 mg l^−1^) Precursor: MVA	20E, polypodine B	Corio-Costet et al. (1998)
*Cyanotis arachnoidea* C.B. Clarke Commelinaceae	Cell suspension	MS with BAP (3.0 mg l^−1^) + NAA (0.2 mg l^−1^) Elicitors: MeJA, AgNO_3_	20E	Wang et al. (2014)
*Polypodium vulgare* L. Polypodiaceae	Prothalli cultures	½ MS Precursors: MVA, cholesterol, ecdysone	Ecdysone, 20E, polypodine B, pterosterone	Reixach et al. (1996)
		½ MS temp. 45 °C		Reixach et al. (1997)
		½ MS with BAP (to 30 mg l^−1^) + 2,4-D (0–3.0 mg l^−1^)		Camps et al. (1990)
*Pteridium aquilinum* L. Kuhn Pteridaceae	Callus and cell suspension	MS with 2,4-D (1.0 μM) + Kin (1.0 μM) or MS with NAA (1.0 μM) + Kin (1.0 μM)	20E, ecdysone, ponasterone, polypodine B	Macek and Vanek (1994)
*Rhaponticum carthamoides* (Willd.) Iljin Asteraceae	Hairy roots	Hormone free SH, WPM and B_5_	20E	Skała et al. (2015)
*Serratula tinctoria* L. Asteraceae	Callus and cell suspension	MS with 2,4-D (5.0 mg l^−1^) or 2,4-D (5.0 mg l^−1^) and BAP (0.2 mg l^−1^)	20E, 20E − 3-acetate, polypodine B	Corio-Costet et al. (1993, 1996)
	Hairy roots	Hormone free MS Precursors: MVA, cholesterol		Delbecque et al. (1995)
		Hormone free MS Precursor: cholesterol		Corio-Costet et al. (1996, 1999)
*Trianthema portulacastrum* L. Aizoaceae	Callus	MS with 2,4-D (2.0 ppm) or 2,4-D (2.0 ppm) and Kin (0.4 ppm)	Ecdysterone	Ravishankar and Mehta (1979)
*Vitex glabrata* R.Br. Lamiaceae	Cell suspensions	B_5_ with BAP (2.0 mg l^−1^) + 2,4-D (1.0 mg l^−1^)	20E	Sinlaparaya et al. (2007)
		B_5_ with 2,4-D (1.0 mg l^−1^) + BAP (2.0 mg l^−1^) Precursors: cholesterol, ergosterol, 7-dehydrocholesterol		Sinlaparaya et al. (2007)
		B_5_ with 2,4-D (1.0 mg l^−1^) + BAP (2.0 mg l^−1^) temp. 25 °C, sucrose (30 or 40 g l^−1^), cholesterol (5.0 mg l^−1^)		Thanonkeo et al. (2011)
		½ MS with 2,4-D (1.0 mg l^−1^) + BAP (2.0 mg l^−1^) Elicitors: chitosan, MeJA		Chamnipa et al. (2012)

*2,4*-*D* 2,4-dichlorophenoxyacetic acid; *20E* 20-hydroxyecdysone *B*_*5*_ Gamborg’s medium; *BAP* 6-benzyloaminopurine; *IAA* indole-3-acetic acid, *IBA* indole-3-butyric acid; *Kin* kinetin; *MeJA* methyl jasmonate; *MS* Murashige and Skoog medium; *MVA* mevalonic acid; *MVL* mevalonic lacton; *NAA* α-naphthalene acetic acid; *SH* Schenk and Hildebrandt medium; *WPM* McCown woody plant medium

The current commercial supply of ecdysteroids relies on their extraction from wild plants or cultivation in the field. This approach is accompanied by obvious difficulties related to agriculture and unstable environment and time and resource consumption, while the yield is relatively low. Ecdysteroids can be classified as phytoalexins, so their yield is dependent on many environmental factors—temperature, seasonal variation and geographic location. Additionally, the ecdysteroid content varies in different organs of the plant. Therefore, both wild and cultivated ecdysteroid-rich plants are morphologically and genetically heterogeneous, which is reflected in variable content and diversity of the desired compounds. Moreover, some of these plants are rare, endangered or protected in some areas. This makes the plant in vitro cultures an ecologically friendly source of biomass for both commercial supply and research on ecdysteroids.

### Micropropagation

The reproducible in vitro propagation protocols have been developed for many medicinally important plant species, including those rich in phytoecdysteroids (Table 2). This technique may be an alternative for plant biomass production, especially such production that is rich in the desired bioactive compounds (Rout et al. 2000; Smetanska 2008).

**Table 2:** Micropropagation of selected ecdosteroid-producing plants

Plant species/Family	Initial explant	Shoot regeneration	Root induction	Plantlets acclimatization	Authors
*Achyranthes aspera* L. Amaranthaceae	Nodal segments Shoot tips	MS + BAP 5.0 mg l^−1^ (11 shoots)	½ MS + IBA 1.0 mg l^−1^	68%	Gnanaraj et al. (2012)
*Achyranthes aspera* L. Amaranthaceae	Nodal segments Shoot tips	MS + BAP 2.0 mg l^−1^ + IAA 1.0 mg l^−1^	½ MS + IBA 1.0 mg l^−1^ (3.4 root/explant)	80%	Parveen et al. (2008)
*Achyranthes aspera* L. Amaranthaceae	Callus	MS + BAP 2.0 mg l^−1^ + NAA 0.5 mg l^−1^	MS + IBA 3.0 mg l^−1^	66.67%	Sen et al. (2014)
*Achyranthes bidentata* Blume Amaranthaceae	Nodal segments Shoot tips	MS + BAP 5.0 mg l^−1^ (10 shoots)	½ MS + IBA 1.0 mg l^−1^	68%	Gnanaraj et al. (2012)
*Achyranthes bidentata* Blume Amaranthaceae	Callus	MS + 2,4-D 0.5 mg l^−1^+, NAA 1.0 mg l^−1^ + IBA 0.1 mg l^−1^ + ZT 0.1 mg l^−1^	MS + 2,4-D + NAA/IBA + ZT	NA	Duan et al. (2012)
*Ajuga bracteosa* Wall ex Benth Lamiaceae	Callus	MS + 5.0 BAP mg l^−1^ + IAA 2.0 mg l^−1^ (41.5 shoots/callus)	MS + IBA 0.5 mg l^−1^ (20 roots/shoot)	82%	Kaul et al. (2013)
*Lychnis flos*-*cuculi* L. Caryophyllaceae	Shoot tips of axenic seedlings	MS + BAP + IAA/NAA (13 shoots)	MS + NAA (100%)	80–100%	Thiem et al. (2013) Maliński et al. (2014)
*Pfaffia glomerata* (Spreng.) Pedersen Amaranthaceae	Nodal segments	MS + BAP 2.22 µM + NAA 2.68 µM + 0.1 µM glucose	NA	NA	Vasconcelos et al. (2014)
*Pfaffia glomerata* (Spreng.) Pedersen Amaranthaceae	Nodal segments	MS (1.4–1.7 shoots)	MS (100%)	100%	Flores et al. (2010)
*Pfaffia tuberosa* (Spreng.) Hicken Amaranthaceae	Nodal segments	MS + TDZ 1.0 µM (10.3 shoots)	MS (100%)	80–100%	Flores et al. (2010)
*Rhaponticum carthamoides* (Willd) Iljin Asteraceae	Callus	MS + 2,4-D 0.25 mg l^−1^ + BAP 1.5 mg l^−1^ (55% shoot/callus)	MS + BAP 0.5 mg l^−1^ + IAA 1.0 mg l^−1^ (100% root/callus)	NA	Zand et al. (2014)
*Vitex negundo* L. Lamiaceae	Nodal segments	MS + BAP 1.0 mg l^−1^ + GA_3_ 0.4 mg l^−1^	MS + IAA 1.0 mg l^−1^ IBA 1.0 mg l^−1^	93%	Sahoo and Chand (1998)
*Vitex negundo* L. Lamiaceae	Nodal explants	BAP 17.8 µM + NAA 2.15 µM + 5% sucrose (20.88 shoots)	IBA 9.4 µM (95.56%)		Chandramu et al. (2003)
*Vitex negundo* L. Lamiaceae	Callus	MS + 2.7 µM TDZ	MS + 1.71 µM IAA + 1.62 µM NAA	70.33–88.71%	Rani and Nair (2006)
*Vitex negundo* L. Lamiaceae	Nodal segments with axillary buds	MS + BAP 1.0 µM + NAA 0.5 µM	Treatment with IBA 500 µM/10 min	95%	Ahmad and Anis (2007)
*Vitex negundo* L. Lamiaceae	Nodal explants	MS + BAP 5.0 µM + 0.5 µM (16 shoots)	MS + IBA 10 µM	97%	Ahmad and Anis (2011)
*Vitex trifolia* L. Lamiaceae	Nodal segments	MS + BAP 5.0 µM + NAA 0.5 µM (6.2 shoots)	Treatment with IBA 500 µM/20 min	95%	Ahmad et al. (2013)

*2,4*-*D* 2,4-dichlorophenoxyacetic acid; *BAP* 6-benzylaminopurine; *GA*_*3*_ gibberrelic acid; *IAA* indole-3-acetic acid; *IBA* indole-3-butyric acid; *MS* Murashige and Skoog medium; *NA* not available; *NAA* α-naphthalene acetic acid; *TDZ* thidiazuron; *ZT* zeatin

*Achyranthes aspera* L. and *A. bidentata* Blume possess valuable medicinal properties. Therefore, attempts were made to amplify the biomass rich in bioactive compounds. The highest percentage of shootlets formation and shoot proliferation of both species were initiated via regeneration from shoot tips and nodal segments cultured on MS medium supplemented with BAP. From all the tested hormonal combinations, the maximum shoot number was observed for the MS medium plus BAP at a relatively high concentration—5.0 mg l^−1^ (*A. aspera* about 11 and *A. bidentata* about 10). In the next step of micropropagation, the highest number of rootlets of both species was observed when shoots grew in salt-reduced MS medium supplemented with 1.0 mg l^−1^ IBA. From those in vitro-regenerated plants, 68% of the rooted shoots were well established in the green house conditions (Gnanaraj et al. 2012). In the same year, the effect of plant growth regulators on callus redifferentiation in *A. bidentata* was presented by the team of Duan (2012). The bud formation from callus was highly influenced by auxins 2,4-D, NAA and IBA. The effect of 2,4-D was also significant for root induction (Duan et al. 2012). The aim of the studies carried out by Sen et al. (2014) was to regenerate the plantlets of *A. aspera* via indirect organogenesis. Among all the tested regulators, the combination of 2.0 mg l^−1^ BAP and 0.5 mg l^−1^ NAA induced significant shoot regeneration. High root formation frequency (82%) and highest number of roots was obtained in the presence of 3.0 mg l^−1^ IBA. The in vitro-regenerated plantlets, when acclimatized, showed 66.67% survival rate (Sen et al. 2014). Previously, there was only a short and vague conference report on *A. aspera* in vitro propagation. The highest shoot proliferation rate for this species was observed for the MS medium supplemented with 1.0 mg l^−1^ BAP and MS with 2.0 mg l^−1^ BAP + 1.0 mg l^−1^ IAA. The most effective medium for root induction was ½ MS enriched with 1.0 mg l^−1^ IBA (3.4 roots per shoot). In this experiment, the regenerated plantlets acclimatized very well with a survival rate of over 80% (Parveen et al. 2008).

*Ajuga bracteosa* Wall ex Benth., a medicinal herb which is also characterized by the presence of ecdysteroids among the various metabolites, has been propagated under in vitro conditions due to the limited distribution and status of the threatened species. The results of the experiment revealed that the highest shoot regeneration percent (100%) was achieved from callus maintained on MS medium enriched with 5.0 mg l^−1^ BAP and 2.0 mg l^−1^ IAA. The average number of shoots per callus culture was 41.4 shoots with an average height of 8.4 cm. In the following experiment, the regenerated shoots were rooted on MS medium with auxin. IBA at the concentration of 0.5 mg l^−1^ induced the maximum number of roots (20). The plantlets survived with the 82% rate (Kaul et al. 2013).

Another species of medicinal plant, *Gomphrena macrocephala* St.-Hol., displays high level of ecdysterone which is the most widely occurring phytoecdysone. The best result of shoot multiplication for this species was achieved for microshoots cultured on MS medium with BAP (5.0–10.0 mg l^−1^) and NAA (0.1 mg l^−1^). The best rooting of regenerated shoots took place on MS supplemented with IBA (10.0 mg l^−1^) or NAA (5.0 mg l^−1^) (Vieira et al. 1994).

*Pfaffia glomerata* (Spreng) Pedersen and *Pfaffia tuberose* (Spreng) Hicken, both species popularly known as Brazilian ginseng with high economic value, have been used in folk medicine. The medical activity is attributed to the compounds including β-ecdysone as the most important phytoecdysteroid. Micropropagation of these two species has been widely described by a Brazilian research group, mostly represented by Flores, who also gathered all the results on shoot proliferation, elongation, rooting, plantlets acclimatization and soil culture in one publication (Flores et al. 2010). Explants (nodal segments) cultivated on MS medium without plant growth regulators were able to regenerate whole plantlets. Addition of TDZ to the medium was not needed for mass propagation, although it was satisfactory for the shoot induction. On the other hand, the presence of this cytokinin inhibited the root growth. In vitro-derived plantlets of both species were successfully transferred to the field conditions (Flores et al. 2010). A study carried out by Vasconcelos et al. evaluated the protocol of in vitro clonal propagation of *Pfaffia glomerata*. The highest shoot proliferation efficiency (35 and 43 shoots per explant) was obtained on MS medium supplemented with 2.22 µM BAP + 2.68 µM NAA and glucose or sucrose at 0.1 M and the multiple shoot multiplication was highly genotype-dependent (Vasconcelos et al. 2014).

A valuable medicinal herb *Rhaponticum carthamoides* (Willd.) Iljin. contains several groups of bioactive compounds, the main of which are ecdysteroids. The regeneration system via callus was established in order to achieve a satisfactory shoot induction ratio, while roots were obtained by direct organogenesis. The maximum regeneration percent was achieved on MS medium with 0.25 mg l^−1^ 2,4-D and 1.5 mg l^−1^ BAP (Zand et al. 2014).

Available information on *Vitex*, a genus of shrubs and trees, includes traditional treatments and clinical potential of many species including *V. negundo* L. and *V. trifolia* L. (Rani and Sharma 2013). An efficient micropropagation method through axillary bud formation was developed for *Vitex negundo*, an important and valuable agro-forestry tree with slow natural regeneration and inefficient propagation through seeds. Under in vitro conditions, the maximum response percentage (97.6) with highest number of shoots (16) was obtained using nodal explants cultured on MS medium with 5.0 µM BAP and 0.5 µM NAA. The best rooting response was achieved on MS supplemented with 10 µM IBA. Finally, the micropropagated plantlets were acclimatized with the rate of 97% (Ahmad and Anis 2011). A few attempts for a direct in vitro regeneration of *V. negundo* were made earlier; however, the results have not satisfied the authors (Sahoo and Chand 1998; Chandramu et al. 2003; Rani and Nair 2006; Ahmad and Anis 2007). As indicated in the experiment, the optimum shoot multiplication and elongation were obtained when TDZ exposed explants were cultured on medium with 1.0 µM BAP and 0.5 µM NAA, whereas the effective root induction was achieved when the shoots were treated with 500 µM IBA for 10 min (Ahmad and Anis 2007). In a different experiment based on the same species (*V. negundo*), the highest shoot induction (97.78%) and multiplication (20.88 per explant) values were observed in the combination treatment with 17.80 µM BAP, 2.15 µM NAA and 5% sucrose. From all the treatments, 4.90 µM IBA added to the media was found to be the most efficient as far as root inducing was concerned (Chandramu et al. 2003). Moreover, the beneficial role of TDZ was noticed for *V. negundo* callus induction and shoot production (Rani and Nair 2006). A high-frequency multiplication rate of *V. negundo* was established on MS with 1.0 mg l^−1^ BAP and 0.4 mg l^−1^ GA_3_ and in vitro-regenerated shoot rooting on MS with IAA and IBA at 1.0 mg l^−1^ (Sahoo and Chand 1998). An in vitro regeneration protocol of *V. trifolia* has been established by testing three cytokinins (BAP, Kin, 2iP) and auxins (IAA, IBA, NAA) in different combinations and concentrations. MS medium enriched with 5.0 µM BAP + 0.5 µM NAA yielded the most effective regeneration (97.33%) and proliferation (6.20). As it turned out, the best rooting of the regenerated shoots occurred when shoots were treated with 500 µM IBA for 20 min. In the next step, a 95% of in vitro-regenerated plantlets survived acclimatization to the field (Ahmad et al. 2013).

The protocol for micropropagation through axillary buds formation has been successfully established for *Lychnis flos*-*cuculi* L., a plant with potential medicinal value. This biotechnological study reports a procedure using shoot tips of axenic seedlings as explants. MS with BA and NAA induced high regeneration efficiency with over 13 shoots per explant. The in vitro regenerated shoots were successfully rooted and transferred into the soil. Preliminary chromatographic analysis by TLC and HPLC indicated that multiple shoots and roots from in vitro-derived plants maintained the ability to accumulate phytoecdysteroids, identified as 20-hydroxyecdysone and polypodine B (Thiem et al. 2013; Maliński et al. 2014; Napierała unpublished).

As it can be read from these studies, the establishment of efficient and reproducible protocols of plant clonal propagation requires the development of a number of parameters associated with the delivery of growth components, especially phytohormones carefully selected in terms of types and concentrations. Receiving a large number of genetically stable plantlets may be essential for obtaining raw plant material with an adjusted content of the desired compounds.

### Factors increasing production

Many physicochemical factors may affect the production of secondary metabolites under laboratory in vitro production. To obtain a high content of bioactive compounds, one must first select a suitable donor plant and induce a properly selected type of culture. The next step is to optimize the culture conditions with high biomass growth and high bioactive compound production, and then select a high-performance line. The accumulation of the compounds may be increased in such a stable plant biomass by the modification of the media composition or the application of biotechnological methods, including elicitation or precursor feeding (Rout et al. 2000; Ramachandra Rao and Ravishankar 2002; Karuppusamy 2009). The most important factors affecting the production of ecdysteroids in in vitro cultures of valuable plant species are described below.

### Tissue and organ cultures for ecdysteroid production

Many authors suggested a huge impact of tissue organization on the production of selected metabolites including ecdysteroids. *Ajuga reptans* characterized by the presence of seven ecdysteroids (ajugalactone, cyasterone, sengosterone, 29-norsengosterone, 29-norcyasterone, 20E and polypodine B) cultured in vitro produced different amount of those compounds, which seemed to be related to differentiation. As it has been demonstrated in extensive biotechnological research, the roots were able to produce ecdysteroids, while those compounds were not detected in the leaves before the differentiation of roots. Moreover, the authors noticed that the content of ecdysteroids increased in the shoot-producing roots. The work has provided irrefutable evidence that ecdysteroids production is root-specific (Tomas et al. 1993). The previous work on *A. reptans* (whole plants and in vitro propagated plantlets, as well as callus cultures) indicated that ecdysteroids production was related to organized structures. Callus obtained from leaves or roots was not able to produce ecdysteroids (Tomas et al. 1992). Similarly, ecdysteroids of *L. flos*-*cuculi* were not detected in callus in contrast to organ cultures (Napierała unpublished).

There exists a known phenomenon of lack of competence of undifferentiated cultures for the production of selected metabolites. In other cases, the cell and callus cultures are characterized by a simplified profile of the compounds. These types of cultures can produce the selected compounds at low concentrations, compared to the organs of intact plants (Corio-Costet et al. 1993a, b, 1996). For example, the analyses of ecdysteroid content in *Serratula tinctoria* (L.) roots showed the presence of polypodine B and high quantities of 20E and its 3-acetate derivative. However, it was found that the cultured cells were able to produce only 20E and, additionally, the compound was present in a low quantity. Generally, the synthesis of ecdysteroids in callus and cell suspension cultures was found to be lower (0.01–0.03% dw) than in the organs of soil-grown plants (0.1–1.2% dw) (Corio-Costet et al. 1993a, 1996). While cell suspension culture of *A. turkestanica* accumulated only 20E, hairy root cultures contained 20E, cyasterone, cyasterone 22-acetate and trace amounts of turkesterone (Cheng et al. 2008).

Another ecdysteroid-rich species, *Chenopodium album* (Fat Hen), was introduced to in vitro cultures. Incorporation of radiolabeled mevalonate into ecdysteroids has shown that cell suspension culture is able to produce them, but at a reduced rate, as compared to intact plants (Corio-Costet et al. 1993b, 1998). An experiment carried out by Filippova et al. (2003) concerning cell suspension and callus cultures of *A. reptans* and *Serratula coronata* has shown that in some cases the yield of ecdysteroids may be higher than in intact plants—in case of *A. reptans* cell suspension cultures, the content of 20E was 4- to 8-fold higher (Filippova et al. 2003).

On the other hand, the analysis of *Pfaffia glomerata* and *P. tuberosa* aerial parts and roots of micropropagated plantlets indicated a significant amount of β-ecdysone in both organs. The shoots of two *P. glomerata* genotypes of wild plants presented 2-fold more β-ecdysone than the roots. Moreover, in vitro-derived plantlets have similar β-ecdysone content as the plants grown in the soil (Flores et al. 2010).

As shown in the presented examples, the production ability of selected compounds and their amounts depend mostly on the cell organization and the type of organ.

### Modification of medium composition

One of the key aspects that concern the production of ecdysteroids is the selection of the optimal medium composition. The nutrients not only allow for the growth of plant tissues and organs, but also they stimulate the biosynthesis of secondary metabolites. A complete culture medium should generally contain macronutrients, micronutrients, vitamins, aminoacids, and a source of carbon (Murashige and Skoog 1962; Gamborg et al. 1968; Lloyd and McCown 1981).

Murashige and Skoog’s medium (MS) contains an optimal nutritional composition for the growth of a large number of plants and cultured tissues, but Gamborg’s medium (B_5_) seems to be suitable for the maintenance of many in vitro cultures as well. For example, Gamborg’s medium was used for the induction of callus from *Ajuga turkestanica* (Regel) Brig. (Cheng et al. 2008). The optimal cell growth and the highest production of 20-hydroxyecdysone (0.038% dw) was observed in the cell suspension of *Vitex glabrata* R.Br. cultured in the Gamborg’s medium. B_5_ medium was 24% more effective than half-strength MS medium, because it is more ample in vitamins and elements (Sinlaparaya et al. 2007). The difference between the MS and Gamborg’s B_5_ media composition has been demonstrated in another experiment, where the content of β-ecdysone in *V. glabrata* cell suspension culture was measured. As in the previously described experiment, the maximum accumulation of cell biomass and 20E content were observed in the B_5_ medium. The authors also noted that in the composition of Gamborg’s medium the amount of nutrients was almost 2-times higher than in ½ MS (Thanonkeo et al. 2011).

Sugar is the main donor of carbon in the medium. Effects of sugar concentration are important for biomass growth and the biosynthesis of metabolites. In plant culture media, the sucrose is used as a carbon source at a concentration of 20–60 g l^−1^. For the highest biomass accumulation and ecdysone synthesis in suspension culture of *V. glabrata*, the optimal concentration of sugar was 30 or 40 g l^−1^, and a lower dose of sugar inhibited cell growth (Thanonkeo et al. 2011).

### Effects of plant growth regulators

The plant hormones are most important for the development of organs, tissues and cells cultured in vitro. The same plant hormones can stimulate growth, cell division and the biosynthesis of secondary metabolites, including ecdysteroids. These are generally classified into the following groups: auxins, cytokinins, gibberellins, and abscissic acid. It is difficult to define which of the groups of growth promoters induce the synthesis of ecdysteroids. The proportion of different types of plant hormones determines the production of secondary metabolites, including ecdysteroids in cells cultured under in vitro conditions.

Auxin has an effect on the induction and growth of *Trianthema portulacastrum* Linn. calli, as well as the production of ecdysterone. The highest concentration of ecdysterone was found in callus growing on a medium supplemented with 2,4-D (2.4 ppm), but the maximum growth of callus was observed in the presence of NAA (0.2 ppm). The increase in the concentration of Kin (cytokinin) inhibited the synthesis of ecdysterone, while the addition of GA_3_ inhibited the growth of callus, while it also stimulated the synthesis of ecdysterone (Ravishankar and Mehta 1979).

On the other hand, the effect of plant growth regulators on the cell growth and 20E production was studied on *Vitex glabrata* suspension culture. The maximum 20E content was obtained in the third week of cultivation in the medium supplemented with 2.0 mg l^−1^ BAP and 1.0 mg l^−1^ 2,4-D and it was 30% higher than in the medium supplemented with 1.0 mg l^−1^ IAA (Sinlaparaya et al. 2007).

Compared with NAA, high concentrations of 2,4-D induced a higher content of ecdysone in callus lines of *Serratula tinctoria* (Corio-Costet et al. 1996). A similar result was obtained for cell suspension cultures of *Vitex glabrata* in which the effects of 2,4–D on ecdysteroids synthesis were better than IAA at equal concentrations (Sinlaparaya et al. 2007). However, high dose of cytokinin (BAP) and low dose or absence of auxins (2,4-D) promoted an increase of β-ecdysone content in in vitro culture of *Polypodium vulgare* (Camps et al. 1990). Optimal biomass growth and quantity of ecdysone was obtained in a medium with NAA and Kin for calli culture of *Pteridium aquilinum* (Macek and Vanek 1994). The phytoecdysteroid production by *Ajuga reptans* in vitro cultures was higher in tissues cultured in media supplemented with plant hormones than in basal media. The addition of NAA to the media strongly increased the phytoecdysteroid production in all types of roots. There was a relationship between biomass growth and ecdysteroid production. All treated roots grew more actively and had an increased ecdysteroid level compared to the control (Tomas et al. 1993).

### The effects of temperature

The temperature of in vitro cultures is an important factor having an influence on cell growth and the progress of phytoecdysteroid biosynthesis.

The research group of Thanonkeo studied the effect of temperature (25 °C and 30 °C) on the biomass growth and 20E production in the suspension culture of *V. glabrata*. The maximum cell growth at 30 °C was 1.06-fold lower than at 25 °C, and the 20E production in the cells cultivated at 25 °C was 1.09-fold higher than at a higher temperature (Thanonkeo et al. 2011).

In another study, the effect of the temperature (25, 45, 50, 55, 60 °C) and time of the action (15, 30, 60 min, then 1, 2, 5, 10 h) on phytoecdysteroid production in *Polypodium vulgare* prothalli from in vitro cultures were investigated. The result of the experiment depended on both the parameters. The highest production of ecdysteroids was detected at 45 °C (10 h). These tested conditions increased the production of the desired compounds from 15 to 23-times, depending on the objectives of the experiment (Reixach et al. 1997).

### Increasing yield by precursor feeding

The biosynthesis of phytoecdysteroids is a multi-step metabolic pathway. Mevalonic acid pathway is directly related to the synthesis of sterol and steroid compounds in plants. Acetic acid, mevalonic acid, cholesterol and its derivatives are the most important precursors. The addition of the precursors is an efficient method allowing an increase in the yield of ecdysteroids in an in vitro culture. The studies on the metabolism of plants show the positive effect of mevalonic acid and cholesterol on the synthesis of the whole family of ecdysteroids. The main tools used were radiolabelled precursors and HPLC-radioactivity detector. Incorporation of radiolabelled cholesterol and mevalonic acid was confirmed in *Serratula tinctoria* hairy roots (Delbecque et al. 1995), *Ajuga reptans* var. *atropurpurea* hairy roots (Fujimoto et al. 2000) and hydroponic culture of *Spinacia oleracea* (Schmelz et al. 2000).

The addition of sodium acetate or mevalonic acid influenced the synthesis of 20-hydroxyecdysone in *Ajuga turkestanica* hairy roots. The level of 20E increased up to 5–10 μg mg^−1^ and the result was better than that obtained by the addition of cholesterol at the same concentration. However, the synthesis of ecdysteroids was not stimulated by these precursors in the cell suspension culture of *A. turkestanica* (Cheng et al. 2008).

The positive influence of a low dose of cholesterol on the cell growth and 20E production was demonstrated in *V. glabrata* cell culture. After 5 days of cultivation, the 20E content reached 33.45 mg 100 g^−1^ dw, which was 1.11-fold higher than in the untreated cells (Thanonkeo et al. 2011).

The feeding of cholesterol at a higher concentration decreased the growth of cells and did not increase the production of 20E by *V. glabrata* cells. Other precursors such as 7-dehydrocholesterol and ergosterol resulted in an increased production of 20E. The maximum 20E amount was found to be 0.045% dw after using 10 mg l^−1^ 7-dehydrocholesterol (the increase was about 1.36-fold above the level obtained for the control cells) and 0.037% dw after using 10 mg l^−1^ ergosterol (1.12-fold increase). It should be mentioned that feeding of 7-dehydrocholesterol and ergosterol did not affect the cell growth (Sinlaparaya et al. 2007).

### Increasing yield by elicitation and stress

The role of many secondary metabolites in plants is to protect the plant in response to environmental stressors. A technique called elicitation is frequently used in plant biotechnology as a means to expose the plant to mid-level stress (eustress). Eliciting a stress response stimulates the biosynthesis of certain secondary metabolites. To evoke such a response, either biotic (yeast extract, chitosan) or abiotic elicitors (UV radiation, elevated or decreased temperature) can be used. Most commonly, however, a plant stress-signaling molecule—methyl jasmonate (MeJA)—is used.

Ecdysteroids fit in the category of substances produced in response to stress, also due to their probable evolutionary role as biochemical warfare against phytophagous insects. An increased level of ecdysteroids has been noted in young parts of *Spinacia oleracea* vulnerable to damage (Bakrim et al. 2008), as well as in physically damaged roots (Schmelz et al. 1998) and in *Polypodium vulgare* prothalli exposed to hot water (Reixach et al. 1997)—which hints the role of ecdysteroids as general stress response molecules that are likely to be playing a protective role.

The suspension cell culture of *V. glabrata* was characterized by a low content of 20E (31.60 mg 100 g^−1^ dw) in the biomass. The elicitors (chitosan and MeJA) were added separately at one out of three different concentrations into the culture medium. The highest value of biomass and production of 20E were obtained when the cells were treated with 50 mg l^−1^ chitosan. The production of 20E (377.09 mg 100 g^−1^ dw) after 8 days of culture increased 8.33-fold above the untreated cells. MeJA at all concentrations increased the accumulation of 20E. The maximum accumulation appeared after four days after the addition of 100 µM MeJA to the culture medium and reached 621.76 mg 100 g^−1^ dw, which was 14.54-fold higher as compared to the control (Chamnipa et al. 2012).

The suspension culture of *Cyanotis arachnoidea*, a plant rich in bioactive phytoecdysteroids, maintained in MS medium with 0.2 mg l^−1^ NAA and 3.0 mg l^−1^ BAP, was characterized by a good cell growth and 20E production (124.14 µg l^−1^). The content of 20E increased 2-, 6- and 8-fold after elicitation with 100 mg l^−1^ YE, 25 µM AgNO_3_ and 0.2 mM MeJA, respectively (Wang et al. 2014).

In another experiment, models for elicitation were hairy root cultures of *Ajuga turkestanica*. The transformed root biomass treated with 125 or 250 µM MeJA produced more 20E than unelicited control. The addition of MeJA to the cell suspension culture of *A. turkestanica* provoked not statistically significant increase in 20E concentration, as compared to the control. The average content of 20E in the control culture was 6.9 µg mg^−1^ dw (Cheng et al. 2008).

Addition of 0.6 mM MeJA for 6 days increased the production of 20E in the cell suspension culture of *Achyranthes bidentata* by about 2.6-fold (Wang et al. 2013).

### Hairy roots (infection with Agrobacterium rhizogenes)

Transformation by *Agrobacterium rhizogenes* is a frequent method used in plant biotechnology. *A. rhizogenes* introduces plasmid (Ri plasmid) that induces formation of hairy roots in dicotyledonous plants. Hairy roots are rapidly proliferating adjective roots growing in media without plant growth regulators under in vitro conditions. The advantages of the hairy root system are: genetic and biochemical stability and the ability to synthesize secondary metabolites at a level even higher than those found in the roots of field-grown plants. Genetic and metabolomic selection of superior clones can ensure high performance production of secondary metabolites (Georgiev et al. 2007; Smetanska 2008).

Several studies have focused on the 20E production by *Ajuga* genus. From over 20 clones of *Ajuga reptans* var. *atropurpurea* hairy roots obtained by the transformation of leaves with *A. rhizogenes* MAFF 03-01724, a rapidly growing and well branching line was selected for 20E production measurement. The content of the bioactive compound was 4-times higher in the hairy root biomass (0.12% dw) than in the roots of intact plants. The authors also concluded that the production of steroids was related to the growth of root biomass (Matsumoto and Tanaka 1991; Tanaka et al. 1999). Other studies showed that the hairy roots of *Ajuga reptans* var. *atropurpurea* produced 20E, cyasterone, isocyasterone and 29 norcyasterone as ecdysteroid constituents (Fujimoto et al. 2000).

Hairy root culture was also obtained from other species of *Ajuga* genus—*A.multiflora* Bunge. To induce hairy root formation, the leaves and petioles of intact plants were transformed by A4 strain of *A.rhizogenes*. The production of 20E was 10-times more effective (6.4 mg g^−1^ dw) in transformed roots than in wild-type roots (Kim et al. 2005).

As reported in numerous publications, *Ajuga turkestanica* produces a rich array of bioactive compounds, including phytoecdysteroids such as turkesterone, 20E, cyasterone, cyasterone 22-acetate, ajugalactone, ajugasterone B, α-ecdysone and ecdysone 2,3-monoacetonide. Hairy roots of *A. turkestanica* obtained by transformation of shoots from in vitro-derived plants with K599 strain, and treated with sodium acetate or mevalonic acid, increased 20E production. The cultures treated with mevalonic acid increase the 20E accumulation up to 21.7 µg mg^−1^ (Cheng et al. 2008).

After the transformation of *Serratula tinctoria* seedling stems with an A4 strain of *A. rhizogenes*, the hairy root cultures were established for phytoecdysteroids production. The plant is well known for containing a very high level of 20E and other phytoecdysteroids in the roots. The ecdysteroid content in hairy roots was found to reach 0.1–0.2% dw and the highest concentration was located in the growing part of meristem (Corio-Costet et al. 1999; Delbecque et al. 1995).

Not all hairy root systems have proven the ability to produce 20E. A wide range of the pharmacological properties of *Rhaponticum carthamoides* is attributed to the presence of bioactive compounds including ecdysteroids, with 20E as the principal component in the roots and rhizomes. Due to an inefficient harvest of the roots from the field-grown plants, the use of hairy root cultures seemed to be a desirable source of plant material rich in ecdysteroids. The independent teams of Orlova and Skała reported that hairy root cultures of *R. carthamoides* (after the transformation of rhizomes of intact plant with *A. rhizogenes* strain A4 and leaves from in vitro-derived shoots with *A. rhizogenes* strain ATCC 15834) were unable to produce ecdysteroids (Orlova et al. 1998; Skała et al. 2015).

### Summary and critical comments on ecdysteroids production in in vitro cultures

The present review highlights the biotechnological aspects on the micropropagation, callus and cell suspension cultures of many rich in ecdysteroids species and the factors increasing the production of those valuable secondary metabolites in different types of in vitro cultures. The results show that the accumulation of ecdysteroids in in vitro cultures of some species has a potential for future commercial utilization. Various biotechnological strategies used to induce secondary metabolite overproduction in plant tissue cultures (e.g. media composition, various elicitors, precursor feeding) had a significant effect on ecdysteroid yield in different types of in vitro cultures of selected ecdysteroid-producing plants. However it should be remembered that generally the biotechnological potential of plant in vitro cultures has not yet been explored due to their serious limitations (Filova 2014). The technology has its origin in the first half of twentieth century and since this time just a few secondary metabolites were produced on commercial scale (Filova 2014). Despite significant experimental progress toward enhancement secondary metabolites production has been made, the automation of the process is still limited. Unfortunately some of the most interesting products are accumulated in very small amounts or not produced in unorganized cells. The economy of the production is the major bottleneck (Verpoorte et al. 2002; Karuppusamy 2009; Filova 2014).

## The extraction and pre-purification of the crude extract

The strategy of purification of ecdysteroids from plant material comprises a multi-step procedure, including extraction, pre-purification and chromatography steps. It presents a unique problem, as the ecdysteroids need to be separated from polyphenols, chlorophyll, lipids, triterpenoids, pigment materials, amino acids and other steroids, as well as from each other. The polar nature of ecdysteroids makes it difficult to separate them from other polar constituents in the plant matrix (Ghosh and Laddha 2006).

The dried and milled herbs and biomass from in vitro cultures are usually extracted using methanol or ethanol at ambient temperature. Lipids from extracts can be removed through solvent partition by *n*-hexane-methanol (Russel et al. 1989)*, n*-butanol-water, *n*-propanol-hexane, light petroleum (Lafont et al. 2000). The pre-purification of the crude extract may be thus conducted using methanol-acetone or ethanol-acetone solution (Hunyadi et al. 2007; Cheng et al. 2008), isobutyl acetate-water (ecdysteroids remain in the water phase) and *n*-butanol-water (ecdysteroids go into the butanol phase) (Kubo et al. 1984, 1985). The choice of the right partition system depends on the nature of ecdysteroids to be isolated and the contaminant to be removed.

The extracts are chromatographed over a silica gel column via flash column chromatography (Hunyadi et al. 2007; Cheng et al. 2008). Ecdysteroids are eluted with a step-gradient of solution, such as methanol in dichloromethane (Hunyadi et al. 2007), methanol in chloroform, ethanol in chloroform (Russel et al. 1989), ethyl-acetate in hexane (Cheng et al. 2008) or reversed-phase chromatography with a step gradient of water in methanol (Russel et al. 1989).

## The isolation of ecdysteroids

### Thin-layer chromatography (TLC)

Normal-phase and reversed-phased TLC on silica plates with solvent system such as chloroform–methanol–water (Cheng et al. 2008), chloroform–ethanol, methanol–water, ethanol–water, acetonitrile–water (Lafont et al. 2000) and many others (Hunyadi et al. 2007; Nowak et al. 2013), have been described to be an efficient way to separate ecdysteroids.

The TLC plates are monitored using UV lamps (254 nm), and there is performed visualization by non-specific colour reactions with anisaldehyde spray reagent (Cheng et al. 2008; Nowak et al. 2013), vanillin-sulfuric acid spray reagent, sulfuric (VI) acid or “specific” reactions with ammonium carbonate (fluorescence induction), 2,4-dinitrophenylhydrazine, triphenyltetrazolium chloride, Folin-Ciocialteu reagent (colour reactions) (Dinan 2001).

Thin layer chromatography combined with mass and tandem mass-spectrometry (TLC–MS; TLC–MS–MS) has been described to be a potential technique enabling the identification of ecdysteroids directly from the crude plant extract (Wilson et al. 1990).

Moreover, a number of enhancements of TLC can be made to increase the achieved resolution and to allow more accurate quantitative measurements like automatic multiple development of TLC (AMDTLC), over-pressurized TLC (OPTLC) and high performance TLC (HPTLC) (Read et al. 1990; Wilson et al. 1990; Lafont et al. 2000).

### High performance liquid chromatography (HPLC)

The isolation of ecdysteroids may be monitored using their UV absorbance to employ an UV detector (a strongly absorbing chromophore λ_max_ = 242 nm, log ε ca.4) during HPLC (Lafont et al. 2000). This is a more widely used technique, because it allows high recovery of pure compounds.

The sample preparation usually consisted of purification on solid-phase extraction cartridges (SPE).

Normal-phase HPLC system generally uses silica columns, but polar-bonded columns can also be used (because of solvent gradients) (Lafont et al. 2000). The mobile phase solvents usually consisted of dichloromethane-isopropanol-water or cyclohexane-isopropanol-water mixtures (Maria et al. 2005; Ho et al. 2008).

The most widely used is the reversed-phase HPLC system on C18-bonded columns with methanol–water mobile phase (isocratic or gradient mode) (Sarker et al. 1998; Lafont et al. 2000). Other mixtures that can be used are dichloromethane-methanol (Sarker et al. 1998), isopropanol–water, acetonitrile–water (Lafont et al. 2000). The important thing is to design a system for polar and non-polar metabolites, because they can both be present in the same sample. Moreover, in the case of polar compounds, it may be significant to use different pHs to obtain modified retention times (Lafont et al. 2000).

There are also some other methods for ecdysteroids isolation, however, they are efficient only on an analytical scale (Morgan et al. 1990; Lafont et al. 1994).

## Perspectives on applications of ecdysteroids

Ecdysteroids were recently found to influence multi-drug resistance which is the ability of cancer cells to actively pump out xenobiotics outside the cell due to overexpression and activity of MDR1 protein. The phenomenon is a problematic obstacle in cancer treatment, as it significantly reduces the effectiveness of chemotherapy. Lipophilic derivatives seem to act as inhibitors of the protein—ecdysteroid acetonides decreased the efflux rate of doxorubicin in cells overexpressing the MDR1 protein under in vitro conditions (Martins et al. 2013). Further research concerning the effect of acetonide or dioxolane derivatives of ecdysteroids on the multi-drug resistance phenomenon is currently investigated and may possibly lead to the invention of an effective MDR inhibitor (Martins et al. 2013).

Several studies reported antioxidant, free radical-scavenging and neuroprotective effects of 20-hydroxyecdysone. Both in vitro oxidative damage and in vivo ischemic injury models were investigated. 20-hydroxyecdysone exhibited a protective effect in rat PC-12 cells (adrenal gland, pheochromocytoma) against cobalt chloride induced cell damage (Hu et al. 2010). The detailed studies confirmed the modulatory effects of 20E on NF-κB and JNK signaling pathways and the inhibition of the caspase-3 activity, ultimately responsible for apoptosis. The destructive intracellular phenomena, such as elevation of calcium levels, disruption of the mitochondrial membrane potential and generation of reactive oxygen species, all have been significantly attenuated by 20-hydroxyecdysone (Hu et al. 2012).

## Concluding remarks

An increasing number of ecdysteroid-related papers reflect growing interest in these secondary metabolites which possess many beneficial pharmacological properties. One of the main problems with commercial application of novel ecdysteroid structures is that their extraction from wild or field-grown plants is difficult. The ecdysteroid content in plants is often too low, unpredictably variable and highly dependent on numerous environmental factors.

Plant in vitro cultures constitute a valuable alternative to field-grown plants, because plant biomass is grown under controlled, widely adjustable laboratory conditions, independent from environmental factors. Moreover, the production of desired secondary metabolites in in vitro cell, tissue and organ cultures may be furtherly increased by using a wide spectrum of long known biotechnological methods—including, but not limited to, elicitation, precursor feeding and hairy root cultures.

To sum up, the progress in the field of plant biotechnology concerning ecdysteroid production may significantly influence the progress in the field of cell biochemistry, providing useful molecular tools to study the phenomena related to many degenerative diseases, providing at the same time valuable adaptogens and, perhaps, paving the way to develop gene-switch systems and artificial hormone systems.

## Abbreviations

2,4-D: 2,4-Dichlorophenoxyacetic acid
20E: 20-Hydroxyecdysone
2iP: N6-(2-isopentyl)adenine
B_5_: Gamborg medium
BAP: 6-Benzylaminopurine
GA_3_: Gibberellic acid
HPLC: High-performance liquid chromatography
IAA: Indole-3-acetic acid
IBA: Indole-3-butyric acid
Kin: Kinetin
MeJA: Methyl jasmonate
MS: Murashige & Skoog medium
NAA: Naphthalene-1-acetic acid
polB: Polypodine B
TDZ: Thidiazurone
TLC: Thin layer chromatography
WPM: Woody plant medium
YE: Yeast extract
